# Cardiac contractility modulation as a novel therapeutic approach in transthyretin amyloid cardiomyopathy to improve eligibility to stabilizer therapy: a case report

**DOI:** 10.1093/ehjcr/ytaf608

**Published:** 2025-11-27

**Authors:** Arancha Díaz Expósito, Alejandro I Pérez Cabeza, Paloma Márquez Camas, Ainhoa Robles Mezcua, Jose Manuel García Pinilla

**Affiliations:** Heart Failure and Inherited Cardiac Diseases Unit, Virgen de la Victoria University Hospital, Campus Universitario de Teatinos s/n, 29010 Málaga, Spain; Biomedical Research Institute of Málaga (IBIMA), C/Severo Ochoa 35, 29590 Málaga, Spain; Heart Failure and Inherited Cardiac Diseases Unit, Virgen de la Victoria University Hospital, Campus Universitario de Teatinos s/n, 29010 Málaga, Spain; Biomedical Research Institute of Málaga (IBIMA), C/Severo Ochoa 35, 29590 Málaga, Spain; Heart Failure and Inherited Cardiac Diseases Unit, Virgen de la Victoria University Hospital, Campus Universitario de Teatinos s/n, 29010 Málaga, Spain; Biomedical Research Institute of Málaga (IBIMA), C/Severo Ochoa 35, 29590 Málaga, Spain; Heart Failure and Inherited Cardiac Diseases Unit, Virgen de la Victoria University Hospital, Campus Universitario de Teatinos s/n, 29010 Málaga, Spain; Biomedical Research Institute of Málaga (IBIMA), C/Severo Ochoa 35, 29590 Málaga, Spain; Heart Failure and Inherited Cardiac Diseases Unit, Virgen de la Victoria University Hospital, Campus Universitario de Teatinos s/n, 29010 Málaga, Spain; Biomedical Research Institute of Málaga (IBIMA), C/Severo Ochoa 35, 29590 Málaga, Spain

**Keywords:** Transthyretin cardiac amyloidosis, Cardiac contractility modulation, Heart failure, Tafamidis Case report

## Abstract

**Background:**

Cardiac transthyretin amyloidosis (ATTR-CM) is an infiltrative cardiomyopathy leading to restrictive physiology and, in advanced stages, systolic dysfunction. Conventional heart failure therapy is often poorly tolerated, and Tafamidis access may be restricted in patients with reduced ejection fraction. Cardiac contractility modulation (CCM) enhances contractility and could represent an alternative in this setting.

**Case summary:**

A 76-year-old man with wild-type transthyretin cardiac amyloidosis (ATTRwt) and mildly reduced left ventricular ejection fraction (LEVF 44%) developed persistent symptoms despite optimized medical therapy and enrolment in a clinical trial. Due to persistent systolic dysfunction (LVEF 43%), he was ineligible for Tafamidis reimbursement. A CCM device was implanted in March 2024, resulting in progressive improvement in LVEF to 54% by February 2025, enabling Tafamidis initiation.

**Conclusion:**

Wild-type transthyretin cardiac amyloidosis is underdiagnosed, and treatment options remain limited, particularly in patients with systolic dysfunction. Cardiac contractility modulation has demonstrated benefit in non-infiltrative cardiomyopathies, but evidence in amyloidosis is scarce. Our case represents the second documented worldwide, showing that CCM may improve ventricular function and clinical status and, importantly, may facilitate access to disease-modifying therapy. This report highlights CCM as a potential bridge strategy in selected patients with ATTR-CM and reduced ejection fraction.

Learning points

- Cardiac contractility modulation (CCM) may be considered a novel therapeutic option in patients with transthyretin amyloid cardiomyopathy and mildly reduced ejection fraction who are ineligible for disease-modifying therapy due to reimbursement criteria.
- In selected patients, CCM can lead to clinically meaningful improvements in systolic function, potentially enabling access to stabilizer therapies such as Tafamidis.

## Introduction

Cardiac transthyretin amyloidosis (ATTR-CM) is an infiltrative disease characterized by extracellular deposits of misfolded protein fibrils, leading to myocardial stiffness with restrictive physiology and impaired systolic function in advanced stages.^1^ Current treatment aims to slow disease progression but does not directly improve cardiac contractility or short-term functional status.^2^ Conventional drugs for heart failure (HF) with reduced ejection fraction (HFrEF) are poorly tolerated and have not demonstrated prognostic benefit.

Cardiac contractility modulation (CCM) is a device-based therapy indicated for selected patients with HF with reduced to mildly reduced ejection fraction [left ventricular ejection fraction (LVEF) 25–45%] who remain symptomatic despite guideline-directed medical therapy (GDMT) and are not candidates for cardiac resynchronization therapy (CRT).^3^ Through non-excitatory electrical impulses delivered during the absolute refractory period, CCM enhances myocardial contractility without increasing oxygen consumption. Its mechanism relies on intracellular calcium modulation, optimizing excitation–contraction coupling in viable myocytes.^4^ While its efficacy has been validated in non-infiltrative cardiomyopathies, CCM could represent an innovative therapeutic alternative to improve cardiac function in the context of ATTR-CM, a condition with limited treatment options.^5,6^

## Summary figure

**Figure:**
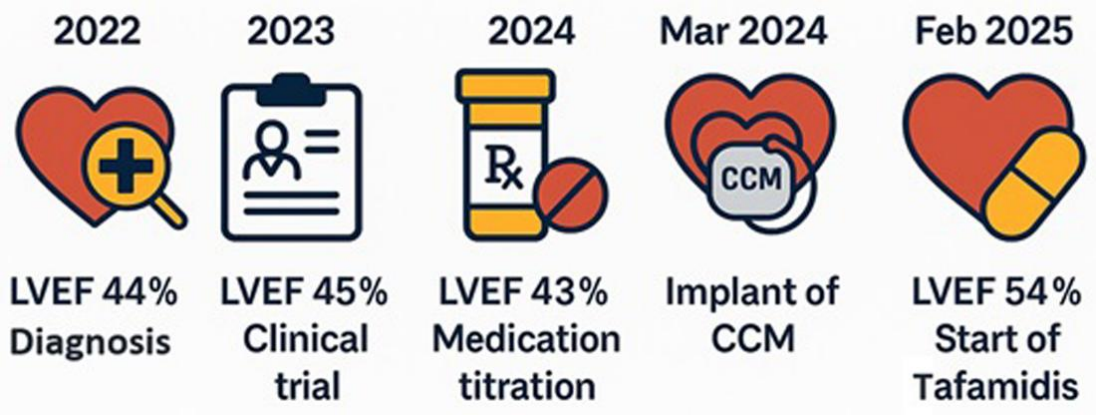


## Case presentation

**Figure 1:**
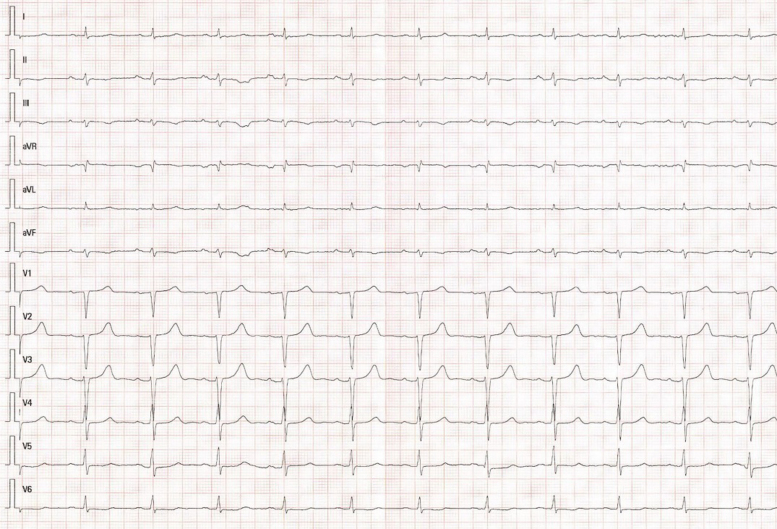
Twelve-lead electrocardiogram showing a pseudo-infarct pattern with Q waves in anterior leads and low QRS voltage.

**Figure 2:**
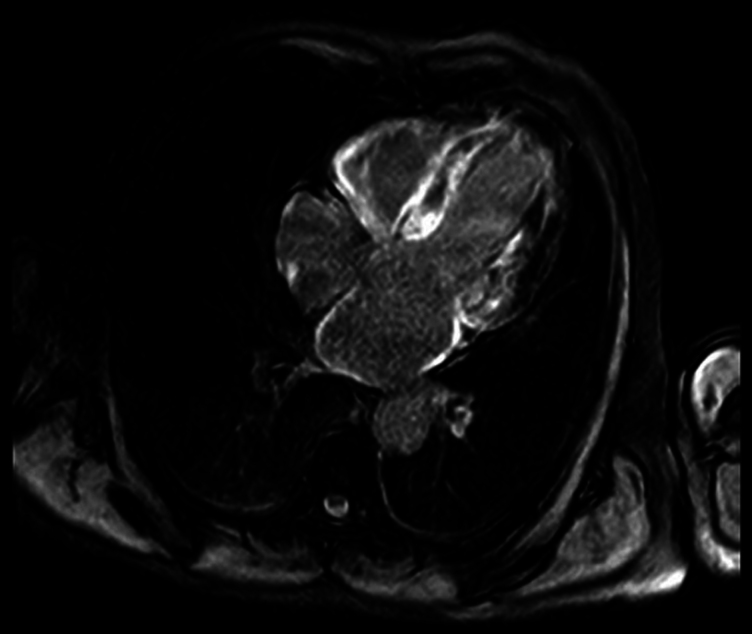
Cardiac magnetic resonance showing diffuse late gadolinium enhancement, more pronounced in the subendocardial layer of the left ventricle, consistent with infiltrative cardiomyopathy. LGE, late gadolinium enhancement.

**Figure 3:**
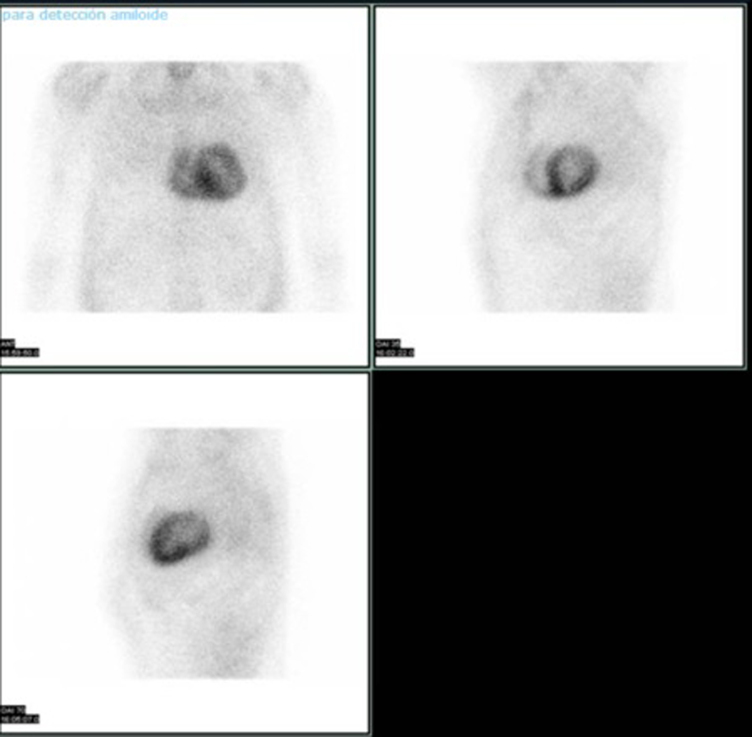
Bone scintigraphy with 99mTc-DPD demonstrating Grade 3 myocardial uptake (Perugini classification).

We present the case of a 76-year-old male who, in December 2021, consulted for progressive asthenia. His medical history included dyslipidaemia, former smoking, and bilateral carpal tunnel syndrome surgery performed 6 months before symptom onset. The initial electrocardiogram showed a pseudo-infarct pattern and low QRS voltage (*Figure 1*). Transthoracic echocardiography (TTE) revealed severe concentric left ventricular hypertrophy with a septal thickness of 17 mm, LVEF of 44%, left ventricular end-diastolic volume (LVEDV) of 91 mL, and left ventricular end-systolic volume (LVESV) of 45 mL and an apical sparing pattern on global longitudinal strain (GLS −9.3%), suggestive of an infiltrative cardiomyopathy. The estimated E/E′ ratio was 24, with biatrial enlargement, normal right ventricular dimensions, a tricuspid annular plane systolic excursion (TAPSE) of 20 mm, and an estimated pulmonary artery systolic pressure (PASP) of 49 mmHg. Cardiac magnetic resonance imaging demonstrated diffuse late gadolinium enhancement, most prominent in the subendocardial region of the left ventricle, with additional involvement of both atria and the right ventricular free wall (Video *1*; *Figure 2*). Bone scintigraphy with 99mTc-DPD showed Grade 3 myocardial uptake (Perugini classification) (*Figure 3*). At that time, his laboratory values were haemoglobin 14.3 g/dL (reference 13.0–17.0 g/dL), creatinine 1.4 mg/dL (reference 0.7–1.3 mg/dL), and N-terminal pro-B-type natriuretic peptide (NTproBNP) 2065 pg/mL (reference < 125 pg/mL). Light chain amyloidosis (AL) was ruled out through serum and urine immunofixation and free light chain analysis, with no monoclonal component detected. Genetic testing for *TTR* mutations revealed no pathogenic variants. Electromyography was normal, with no findings suggestive of neurological involvement. Based on these findings, a diagnosis of wild-type transthyretin cardiac amyloidosis (ATTRwt) with extracardiac involvement was made.

Due to therapeutic limitations at the time, the patient was enrolled in the CARDIO-TTransform trial (NCT04136171), a randomized, double-blind, placebo-controlled study evaluating the efficacy and safety of eplontersen in patients with ATTR-CM. During follow-up in April 2023, echocardiography showed a stable LVEF of 45% and a NTproBNP level of 1723 pg/mL, and cardiopulmonary exercise testing revealed a peak oxygen uptake (VO₂) of 17.8 mL/kg/min (74% predicted) and ventilatory equivalent for carbon dioxide (VE/VCO₂) slope of 34. As there was no significant structural or functional improvement, and to optimize the clinical condition prior to disease-modifying therapy, sequential pharmacologic treatment with ramipril, eplerenone, bisoprolol, and dapagliflozin was initiated and gradually up-titrated over the following months. By September 2023, maximum tolerated doses were achieved (ramipril 2.5 mg, eplerenone 25 mg, bisoprolol 1.25 mg, and dapagliflozin 10 mg), which were maintained thereafter. The patient continuously received furosemide 40 mg/day throughout follow-up, with no need for dose escalation. However, in February 2024, a follow-up echocardiogram showed persistent ventricular dysfunction with an LVEF of 43%.

The implantation of a CCM device was agreed upon and performed in March 2024 without complications. In subsequent follow-ups, progressive improvement in ventricular function was observed, with an LVEF of 47% in June 2024 and 54% in February 2025 (Video *2*), allowing for approval and initiation of Tafamidis 61 mg. In this most recent follow-up TTE, diastolic parameters showed a trend towards improvement (E/E´ ratio 22) together with a reduction in PASP to 44 mmHg and a stable NTproBNP level of 1812pg/mL. These findings suggested a tendency towards better diastolic function, although without significant changes compared with previous echocardiographic assessments, and they could partly reflect the inherent variability between serial examinations, along with an improvement in the patient’s functional class, under stable diuretic therapy.

## Discussion

Wild-type transthyretin cardiac amyloidosis is an underdiagnosed condition whose prevalence increases with age and may clinically present as HF with preserved ejection fraction or with systolic dysfunction in more advanced stages.^1,2,7^ Although Tafamidis has been shown to reduce mortality and hospitalizations in patients with ATTR, in Spain, its reimbursement is currently limited to those with preserved ejection fraction (LVEF > 50%), excluding a significant subgroup of symptomatic patients.^8^

Cardiac contractility modulation is a device-based therapy that has shown efficacy in improving functional class, quality of life, exercise capacity, and, in some cases, LVEF in patients with reduced to mildly reduced ejection fraction and narrow QRS who are not candidates for CRT.^9,10^ It works by delivering high-amplitude electrical impulses during the absolute refractory period, which do not trigger myocardial contraction but do modulate key cellular processes. These include improvement of intracellular calcium homeostasis through increased phosphorylation of phospholamban and activation of the sarcoplasmic/endoplasmic reticulum Ca^2+^-ATPase (SERCA2a), promoting enhanced calcium reuptake into the sarcoplasmic reticulum.^10,11^ Cardiac contractility modulation also partially reverses foetal gene expression, modulates signalling pathways such as phosphoinositide 3-kinase (PI3 K)/protein kinase B(PKB) and mitogen-activated protein kinase (MAPK), and may attenuate fibrosis and pathological remodelling.^10,12^ In the setting of ATTRwt, CCM may hypothetically enhance contractility in functional myocytes, improve excitation–contraction coupling, and reduce amyloid-induced intramyocardial dyssynchrony.

Although most CCM studies have been conducted in patients with non-infiltrative cardiomyopathies, only one published case to date has reported its use in a patient with wild-type transthyretin amyloidosis. Marchese *et al*. described the favourable outcome of a male patient with an LVEF of 38%, intolerance to conventional HF therapies, and recurrent hospitalizations. After CCM device implantation, the patient experienced progressive improvement in functional capacity and ventricular function and remained free from decompensations for over a year.^13^ In addition, the AMY-CCM registry is currently underway, aiming to systematically evaluate the safety and efficacy of CCM in patients with cardiac amyloidosis, reflecting growing clinical interest in this emerging indication (AMY-CCM: ClinicalTrials.gov Identifier: NCT05167799).

Our case represents the second documented experience in the international literature and provides further evidence of the potential benefit of CCM in patients with ATTRwt and symptomatic systolic HF. Notably, the observed improvement in LVEF (from 43% to 54%) allowed for Tafamidis reimbursement and initiation. In this context, CCM may be considered a novel strategy in a disease characterized by poor tolerance to conventional HFrEF medications and limited proven benefit, potentially serving as a bridge in selected patients to facilitate clinical stabilization and echocardiographic improvement, ultimately enabling access to disease-modifying therapies. 

## Lead author biography


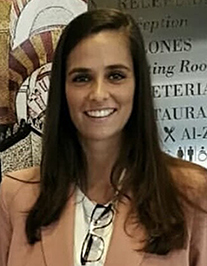


Arancha Díaz Expósito is a Cardiologist at Hospital Virgen de la Victoria in Málaga, specializing in inherited cardiomyopathies and HF. She combines clinical practice with research activity through a Río Hortega Fellowship, contributing to multiple projects in the field of familial cardiac disease. She has co-authored publications and presented at national and international conferences.

## Data Availability

All data underlying this article are included in the manuscript. No additional data are available.
